# Machine learning predicts lymph node metastasis of poorly differentiated-type intramucosal gastric cancer

**DOI:** 10.1038/s41598-020-80582-w

**Published:** 2021-01-14

**Authors:** Cheng-Mao Zhou, Ying Wang, Hao-Tian Ye, Shuping Yan, Muhuo Ji, Panmiao Liu, Jian-Jun Yang

**Affiliations:** 1 Department of Anesthesiology, Pain and Perioperative Medicine, The First Affiliated Hospital of Zhengzhou University, Zhengzhou, Henan China; 2 Department of Pathology, The First Affiliated Hospital of Zhengzhou University, Zhengzhou, Henan China

**Keywords:** Cancer epidemiology, Cancer genetics, Cancer therapy, Tumour biomarkers, Biomarkers, Diseases, Health care, Medical research, Risk factors, Energy science and technology

## Abstract

To construct a machine learning algorithm model of lymph node metastasis (LNM) in patients with poorly differentiated-type intramucosal gastric cancer. 1169 patients with postoperative gastric cancer were divided into a training group and a test group at a ratio of 7:3. The model for lymph node metastasis was established with python machine learning. The Gbdt algorithm in the machine learning results finds that number of resected nodes, lymphovascular invasion and tumor size are the primary 3 factors that account for the weight of LNM. Effect of the LNM model of PDC gastric cancer patients in the training group: Among the 7 algorithm models, the highest accuracy rate was that of GBDT (0.955); The AUC values for the 7 algorithms were, from high to low, XGB (0.881), RF (0.802), GBDT (0.798), LR (0.778), XGB + LR (0.739), RF + LR (0.691) and GBDT + LR (0.626). Results of the LNM model of PDC gastric cancer patients in test group : Among the 7 algorithmic models, XGB had the highest accuracy rate (0.952); Among the 7 algorithms, the AUC values, from high to low, were GBDT (0.788), RF (0.765), XGB (0.762), LR (0.750), RF + LR (0.678), GBDT + LR (0.650) and XGB + LR (0.619). Single machine learning algorithm can predict LNM in poorly differentiated-type intramucosal gastric cancer, but fusion algorithm can not improve the effect of machine learning in predicting LNM.

## Introduction

Gastric cancer is the world's fourth most common neoplastic disease, and the second most fatal tumor-related disease^1^. With the development of endoscopic techniques, improved diagnostics and the global popularization of gastric cancer screening, the early gastric cancer (EGC) detection rate increases every year, especially in Japan and Korea^2,3^. EGC can be treated with endoscopic resection, D1 or D2 radical surgical resection, as well as other medical auxiliary treatments according to tumor stage^4^. The indications and effects of the various treatments vary. EGC only considers the depth of focal infiltration; it does not consider lymph node metastasis, an important factor in choosing an EGC treatment regimen. Therefore, it is necessary to accurately stage EGC patients prior to surgery to select a reasonable treatment option. Studies have shown that EGC with lymph node metastasis (LNM), the number of lymph node metastases, and lymph node metastasis in different regions, have important effects on EGC treatment and prognosis^5^. Therefore, for over 80% of patients with EGC, radical surgery on D1 or D2 increases unnecessary lymph node dissection. It also increases the trauma caused by surgery, and affects patient recovery. In recent years, the development of endoscopic mucosal dissection and endoscopic mucosal resection has brought new developments to EGC treatment. There is now less trauma and quick postoperative recovery. Thus, patients can avoid the heavy trauma and long recovery time caused by laparotomy or endoscopic surgery. However, it is important to accurately judge lymph node metastasis before surgery^6^.

In recent years, many studies have reported on machine learning in medicine. For example, using large preoperative data to develop and validate machine learning algorithms can predict hospital stay and patient-specific hospital costs after primary total hip arthroplasty^7^; Additionally, machine learning can predict hospital acquired pneumonia in patients with schizophrenia^8^; Machine learning techniques can also predict 5-year survival in patients with chondrosarcoma^9^.

However, few studies have investigated the prediction of LNM in early poorly differentiated early gastric cancer^10–12^. This study assesses clinicopathological factors for predicting LNM in intramucosal PDC. It also develops and validates a risk model for predicting LNM using machine learning to provide a basis for the treatment of poorly differentiated-type intramucosal gastric cancer.

## Methods

### Study population

There were no human involved in this study. And this is only a secondary data analysis study using public databases. Data are available from the BioStudies (public) database (https://www.ebi.ac.uk/biostudies/studies?query=S-EPMC4881979), accession numbers: S-EPMC4881979. We prospectively analyzed data from patients diagnosed with PDC who had undergone radical gastrorectal resection and lymph node dissection. Patients included in the study were confirmed as having pure poor differentiated-type T1 (tumor invasion confined to mucosa or submucosa) gastric cancers. The tumors were classified histologically according to the World Health Organization’s Classification of Tumors^13^.

### Analysis of clinical results

The following clinicopathological factors were included in the study, including, presence of lymphangitic involvement (LVI), gender, tumor depth, age, presence of ulcer, tumor size, location of tumor, general appearance, number of resected nodules and presence of LNM. Tumors were staged according to the Seventh Edition of the American Joint Committee on Cancer Staging (7th Edition)^14^.

### Machine learning

Logistic regression (LR) is a broad classification machine algorithm that can predict the probability of future results, whereas "regression" is actually a classification. Accurate, logistic regression is a dichotomous classification algorithm.

Random forest (RF) is a supervised learning algorithm. It is trained with the "bagging" method. The bagging method combines multiple models, and can be more effective than a single model. Thus, it can increase the overall effect.

XGB generates multiple regression trees based on features, and each regression tree learns the corresponding residuals, and the sum of the residuals is the predicted value of the sample.

GBDT is an integrated learning method that uses gradients as input to later trees to learn multiple trees. The combination of multiple trees can then generate a comprehensive learner with strong generalizability.

### Statistical analysis

Statistical analysis was conducted in R, version 3.4.3(https://cran.r-project.org/bin/windows/base/old/3.4.3/), and machine learning modeling was performed with python, version 3.6.5 (https://www.python.org/downloads/release/python-365/). Pearson’s correlation analysis was calculated, and the machine learning algorithm was performed with the following algorithms: XGB, RF, GBDT, LR, XGB + LR, RF + LR and GBDT + LR. 70% of the data was divided into training groups for development, and 30% were verified by the test groups. When missing values were dichotomous, the number of digits was used, and multiple imputation was used for continuous variables. MSE refers to mean squared error.

### Ethics approval and consent to participate

This was a secondary data analysis study using data from the BioStudies public database.

## Results

A total of 1169 patients were enrolled, with lymph node metastases occurring in 61 (5.2%) of them. The age of the lymph node metastasis and non-metastasis groups did not statistically vary between the training and test groups (*P* = 0.281 and *P* = 0.115, respectively) (see Table 1).

**Table 1:** Patient basic characteristic information.

LNM	Training			Test		
	No	Yes	*P* value	No	Yes	*P* value
N	775	43		333	18	
Age (years)	53.0 ± 10.8	50.9 ± 10.3	0.281	52.8 ± 11.1	48.1 ± 11.9	0.115
Tumor size (cm)	2.9 ± 2.0	4.4 ± 2.6	< 0.001	3.1 ± 1.9	3.5 ± 2.2	0.433
Number of resected nodes	40.2 ± 13.6	46.2 ± 14.7	0.009	40.6 ± 13.9	40.4 ± 12.3	0.918
Sex			-			0.630
Female	353 (45.5%)	29 (67.4%)		144 (43.2%)	9 (50.0%)	
Male	422 (54.5%)	14 (32.6%)		189 (56.8%)	9 (50.0%)	
Tumor location			0.598			0.561
Lower	363 (46.8%)	18 (41.9%)		152 (45.6%)	10 (55.6%)	
Middle	335 (43.2%)	19 (44.2%)		156 (46.8%)	8 (44.4%)	
Upper	77 (9.9%)	6 (14.0%)		25 (7.5%)	0 (0.0%)	
Macroscopic type			0.691			0.347
Depressed	415 (53.5%)	24 (55.8%)		170 (51.1%)	9 (50.0%)	
Flat	152 (19.6%)	7 (16.3%)		61 (18.3%)	1 (5.6%)	
Elevated	18 (2.3%)	0 (0.0%)		6 (1.8%)	0 (0.0%)	
Mixed	190 (24.5%)	12 (27.9%)		96 (28.8%)	8 (44.4%)	
Depth of invasion			0.007			0.017
Lamina propria	282 (36.4%)	7 (16.3%)		105 (31.5%)	1 (5.6%)	
Muscularis mucosa	493 (63.6%)	36 (83.7%)		228 (68.5%)	17 (94.4%)	
ymphatic-vascular involvement			< 0.001			< 0.001
No	760 (98.1%)	36 (83.7%)		323 (97.0%)	13 (72.2%)	
Yes	15 (1.9%)	7 (16.3%)		10 (3.0%)	5 (27.8%)	
Ulcer			0.271			0.619
No	737 (95.1%)	39 (90.7%)		310 (93.1%)	18 (100.0%)	
Yes	38 (4.9%)	4 (9.3%)		23 (6.9%)	0 (0.0%)	

**Figure 1:**
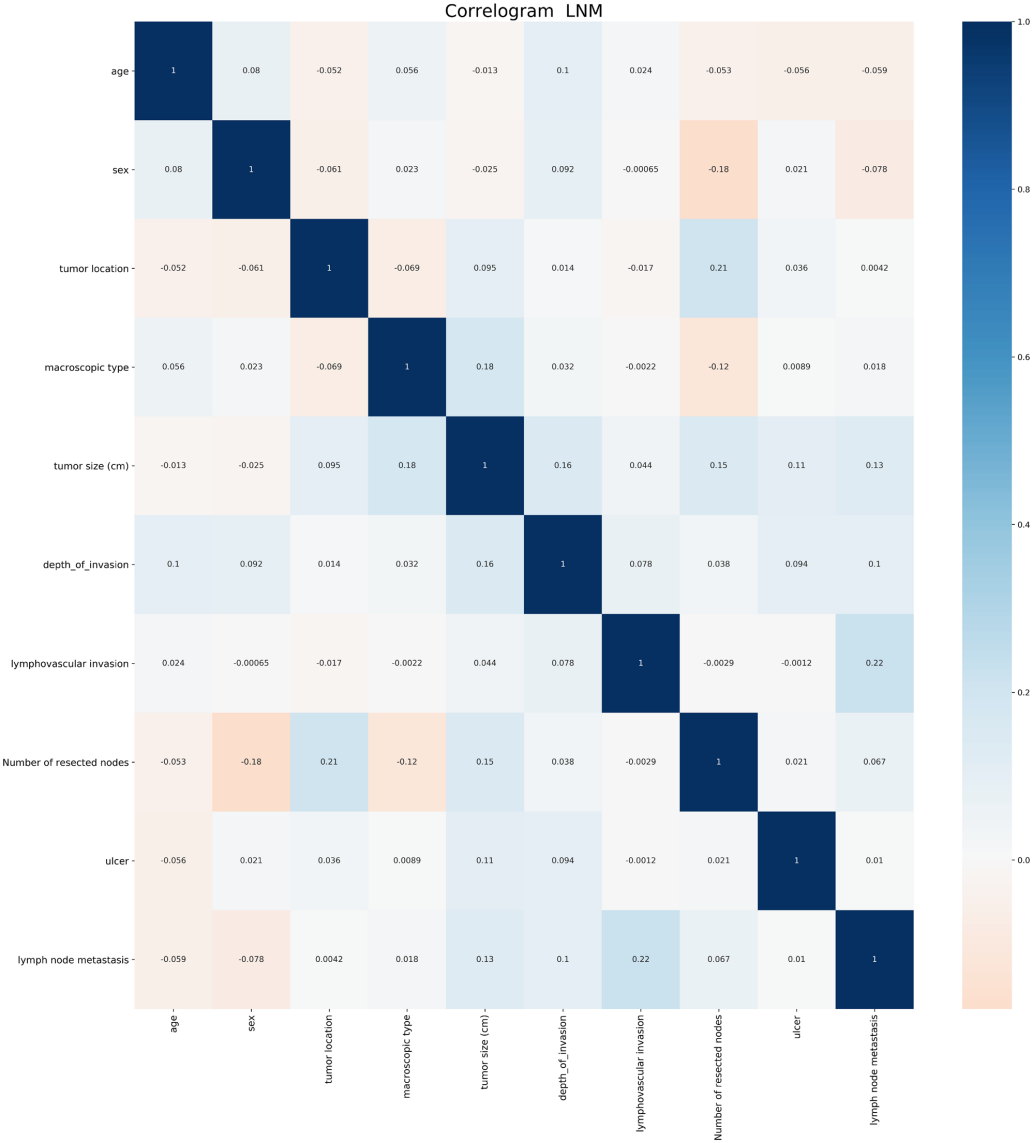
Correlation between factors.

**Figure 2:**
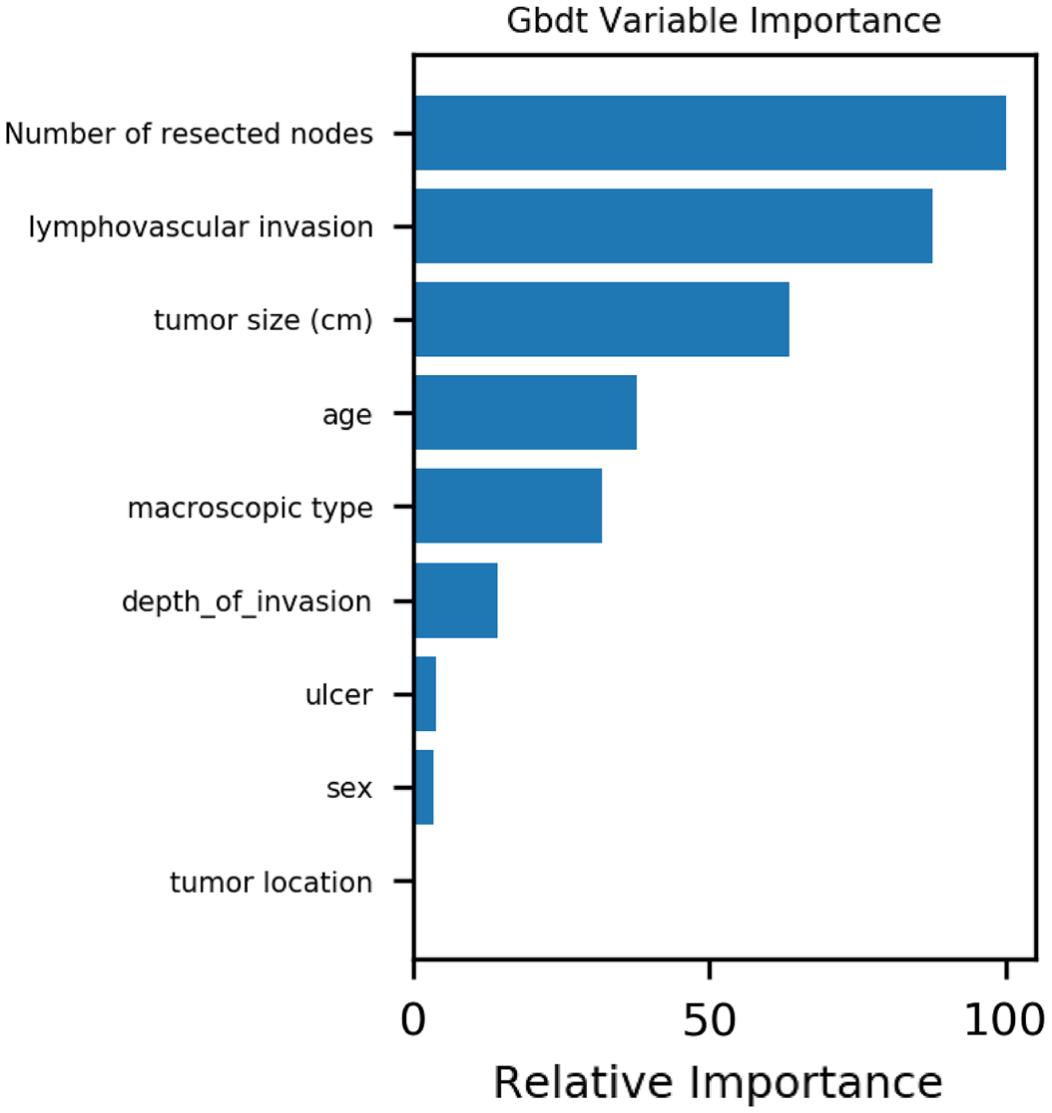
Variable importance of features included in machine learning algorithm for prediction of LNM.

Correlation analysis showed that lymph node invasion, tumor invasion depth, and tumor size were positively correlated with LNM (Fig. 1). In addition, the Gbdt algorithm in the machine learning results finds that number of resected nodes, lymphovascular invasion and tumor size are the primary 3 factors that account for the weight of LNM (see Fig. 2).

**Figure 3:**
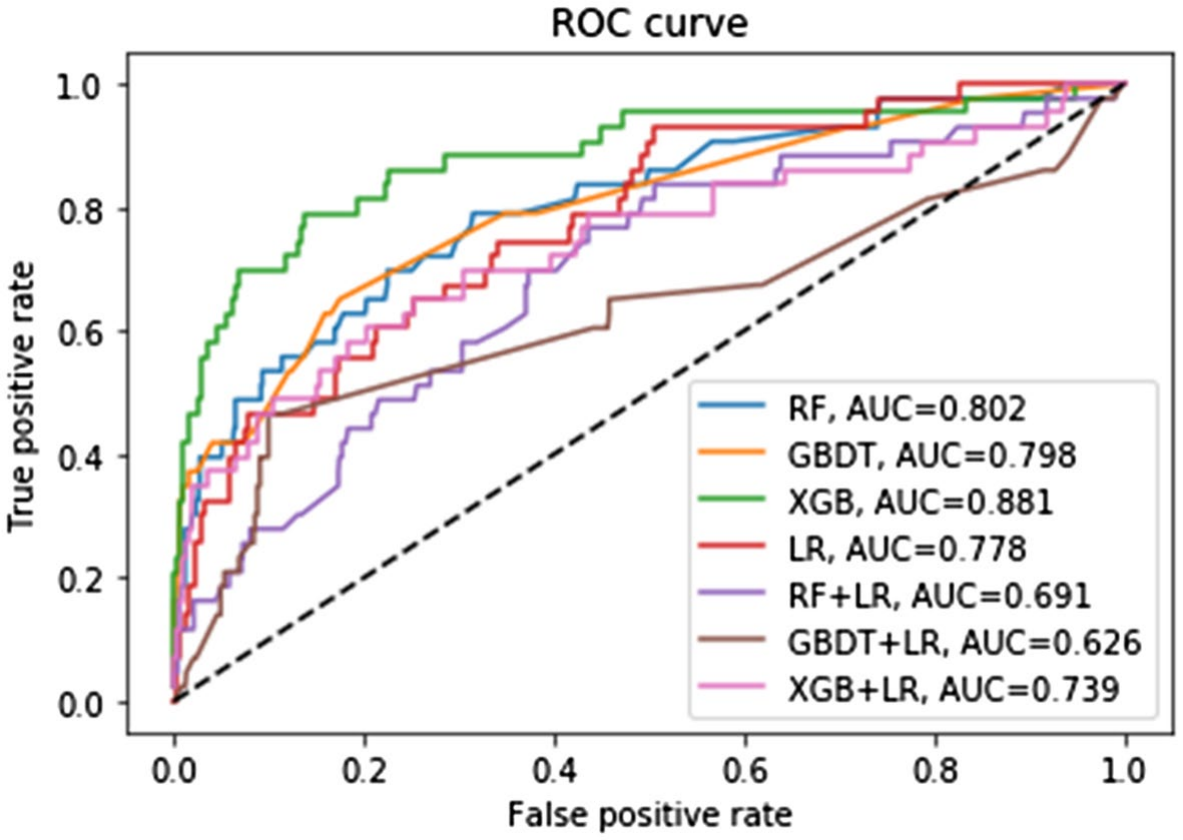
Different machine learning algorithms predict the LNM in the training group.

Effect of the LNM model of PDC gastric cancer patients in the training group: Among the 7 algorithm models, the highest accuracy rate was that of GBDT (0.955); The AUC values for the 7 algorithms were, from high to low, XGB (0.881), RF (0.802), GBDT (0.798), LR (0.778), XGB + LR (0.739), RF + LR (0.691) and GBDT + LR (0.626). Among the 7 algorithms, GBDT’s MSE was the lowest (0.045) and LR was the highest (0.054) (see Table 2 and Fig. 3).

**Table 2:** Forecast results for training and test group.

	Training			Test		
	Accuracy	AUC	MSE	Accuracy	AUC	MSE
RF	0.947	0.802	0.053	0.949	0.765	0.051
GBDT	0.955	0.798	0.045	0.946	0.788	0.054
XGB	0.949	0.881	0.051	0.952	0.762	0.048
LR	0.946	0.778	0.054	0.946	0.750	0.054
RF + LR	0.947	0.691	0.053	0.949	0.678	0.051
GBDT + LR	0.947	0.626	0.053	0.949	0.650	0.051
XGB + LR	0.951	0.739	0.049	0.946	0.619	0.054

**Figure 4:**
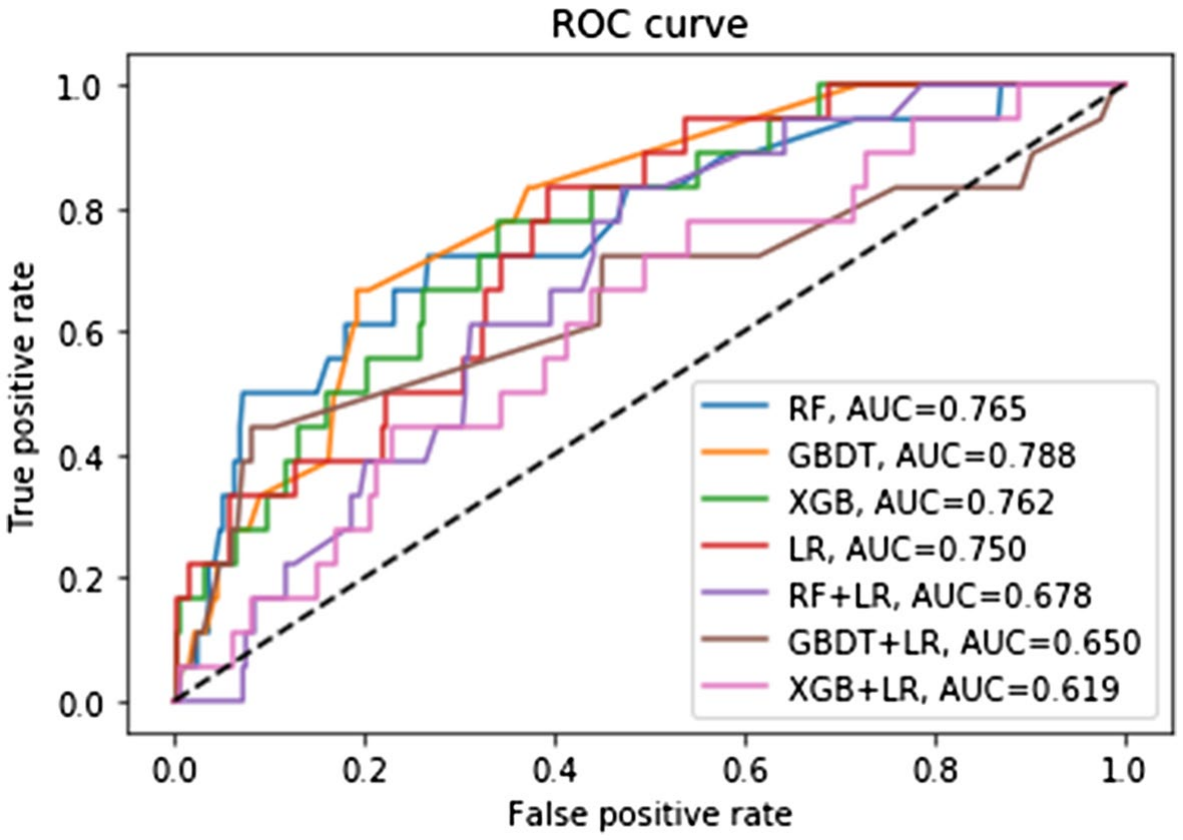
Different machine learning algorithms predict the LNM in the test group.

Results of the LNM model of PDC gastric cancer patients in test group: Among the 7 algorithmic models, XGB had the highest accuracy rate (0.952); Among the 7 algorithms, the AUC values, from high to low, were GBDT (0.788), RF (0.765), XGB (0.762), LR (0.750), RF + LR (0.678), GBDT + LR (0.650) and XGB + LR (0.619). XGB had the lowest MSE (0.048) (see Table 2 and Fig. 4).

## Discussion

At present, research has focused on minimally invasive surgery that can maintain postoperative patient survival rates. The goal is to minimize surgical injury with safe and effective operating procedures, so that patients can enjoy higher quality of life^15,16^. The incidence of lymph node metastasis has been reported to be between 2.2 and 4.2% for intramucosal (T1a) primary gastric adenocarcinoma, and between 9.4 and 16.1% for early (T1) primary gastric adenocarcinoma^10,12^. Our findings suggest that 5.2% of patients with poorly differentiated-type intramucosal gastric cancer develop lymph node metastases. This is consistent with previous findings. Furthermore, the results of this study indicate that the Gbdt machine learning algorithm yields the first 3 factors that account for the weight of lymph node metastasis: number of resected nodes, lymphovascular invasion and tumor size. At the same time, single machine learning algorithm can predict LNM in poorly differentiated-type intramucosal gastric cancer, but fusion algorithm can not improve the effect of machine learning in predicting LNM.

Many clinical pathological factors related to LNM in early gastric cancer have been studied^17,18^. A large sample study in the United States showed that tumor stage, pathological type, and tumor size are independent predictors of LNMin early gastric cancer^19^. Chen et al. have concluded that tumor diameter ≥ 3 cm, whether it is pathological or low-differentiation type, whether it is mixed adenocarcinoma or signet ring cell carcinoma, tumor infiltration into the submucosa, and vascular invasion are independent risk factors for LNM^20^. Our results corroborate this view.

The Japanese gastric cancer assistance group noted that the LNM rate was low for tumors > 2 cm in diameter, patients with no ulcers, tumors ≤ 3 cm in diameter, and differentiated intramucosal cancers with ulcers. This could serve as an absolute indication for ESD^21^. Pokala et al. concluded that early intramucosal gastric cancer with tumor diameter < 4 cm has a low risk of LNM, and can be locally resected^22^. This is consistent with the results of our study. Our results corroborate this view.

Submucosal cancers have a higher rate of LNM than intramucosal cancers. Furthermore, they may be rich in capillaries in the submucosa of the gastric wall, which are usucaptible to cancer cell invasion^23,24^. Studies have shown a high rate of LNM in undifferentiated early gastric cancer^25^. As the tumor grows, the invasion deepens and the LNM rate increases. The LNM rate has been shown to be associated with lymphangitic tumor thrombus^26^. Female patients with early gastric cancer are more likely to develop lymph node metastases than males. This is presumably related to endogenous estrogen levels^27^. Another study has shown that low differentiation, infiltration into the submucosa, large tumors, and venous or lymphatic invasion are independent risk factors for LNM^28^. These findings are also corroborated by our findings.

At present, the main problem of machine learning method in medical practice is the lack of application scenarios and related clinical data. At present, a large number of published machine learning articles only use simple machine learning algorithms. In this study, we also use the machine learning fusion algorithm. However, the results of the test set fusion machine learning algorithm are not ideal. This also proves that when the machine learning algorithm is applied in medical clinic, it should pay attention to the application scenarios and the collection of relevant data.

This study has several limitations. Firstly, it only used routine hematoxylin and eosin staining. Therefore, accurate diagnosis of lymph node micrometastases was difficult. For example, lymph node micrometastasis may be a key causative factor in recurrent gastric cancer treatment. Furthermore, this study included only data on tumor characteristics; no data on patient-related tumor genes were collected. This may have contributed to the lack of optimal predictive results. Because different regions, different races and different treatment schemes may cause different incidence of lymphatic metastasis, and the rate of lymph node metastasis in intramucosal gastric adenocarcinoma is low in this study and previous studies.However, these will not affect the prediction results of machine learning in this study. However, more multi-center and forward-looking research is needed in the future.

## Conclusion

Single machine learning algorithm can predict LNM in poorly differentiated-type intramucosal gastric cancer, but fusion algorithm can not improve the effect of machine learning in predicting LNM. This may provide guidance for personalized treatment of such patients.

## Data Availability

Data are available from the BioStudies (public) database (https://www.ebi.ac.uk/biostudies/studies?query=S-EPMC4881979), accession numbers: S-EPMC4881979.

## References

[1] Siegel RL, Miller KD, Jemal A (2018). Cancer statistics, 2018. CA Cancer J. Clin..

[2] Pasechnikov V (2014). Gastric cancer: prevention, screening and early diagnosis. World J. Gastroenterol..

[3] Yu HY (2015). Magnifying narrow-band imaging endoscopy is superior in diagnosis of early gastric cancer. World J. Gastroenterol..

[4] Espinel J (2015). Treatment modalities for early gastric cancer. World J. Gastrointest. Endosc..

[5] Zhao BW (2015). Lymph node metastasis, a unique independent prognostic factor in early gastric cancer. PLoS ONE.

[6] Guo TJ (2015). Feasible endoscopic therapy for early gastric cancer. World J. Gastroenterol..

[7] Ramkumar P (2019). Development and validation of a machine learning algorithm after primary total hip arthroplasty: applications to length of stay and payment models. J. Arthroplasty.

[8] Kuo K (2019). Predicting hospital-acquired pneumonia among schizophrenic patients: a machine learning approach. BMC Med. Inf. Decis. Mak..

[9] Thio Q (2018). Can machine-learning techniques be used for 5-year survival prediction of patients with chondrosarcoma?. Clin. Orthop. Relat. Res..

[10] Lee JH (2012). Predictive factors for lymph node metastasis in patients with poorly differentiated early gastric cancer. Br. J. Surg..

[11] Kim H (2011). Early gastric cancer of signet ring cell carcinoma is more amenable to endoscopic treatment than is early gastric cancer of poorly differentiated tubular adenocarcinoma in select tumor conditions. Surg. Endosc..

[12] Kunisaki C (2009). Risk factors for lymph node metastasis in histologically poorly differentiated type early gastric cancer. Endoscopy.

[13] *World Health Organization Classification of Tumors* (Lyon: IARC Press, 2000)

[14] Kleihues P, Sobin LH (2000). World Health Organization Classification of Tumors. Cancer.

[15] Lee J (2014). Clinical practice guidelines for gastric cancer in Korea: an evidence-based approach. J. Gastric Cancer.

[16] Tanabe S (2017). Gastric cancer treated by endoscopic submucosal dissection or endoscopic mucosal resection in Japan from 2004 through 2006: JGCA nationwide registry conducted in 2013. Gastric Cancer.

[17] Pyo J (2017). Early gastric cancer with a mixed-type Lauren classification is more aggressive and exhibits greater lymph node metastasis. J. Gastroenterol..

[18] Hatta W (2017). A scoring system to stratify curability after endoscopic submucosal dissection for early gastric cancer: "eCura system". Am. J. Gastroenterol..

[19] Pokala S (2018). Lymph node metastasis in early gastric adenocarcinoma in the United States of America. Endoscopy.

[20] Chen L (2017). Risk factors of lymph node metastasis in 1620 early gastric carcinoma radical resections in Jiangsu Province in China: a multicenter clinicopathological study. J. Dig. Dis..

[21] Hasuike N (2019). A non-randomized confirmatory trial of an expanded indication for endoscopic submucosal dissection for intestinal-type gastric cancer (cT1a): the Japan Clinical Oncology Group study (JCOG0607). Gastric Cancer.

[22] Pokala S (2018). Lymph node metastasis in early gastric adenocarcinoma in the United States of America. Endoscopy..

[23] Catalano F (2009). The modern treatment of early gastric cancer: our experience in an Italian cohor. Surg. Endosc..

[24] Ye B (2008). Predictive factors for lymph node metastasis and endoscopic treatment strategies for undifferentiated early gastric cancer. J. Gastroenterol. Hepatol..

[25] Hirasawa T (2009). Incidence of lymph node metastasis and the feasibility of endoscopic resection for undifferentiated-type early gastric cancer. Gastric Cancer.

[26] Kim D (2004). Factors related to lymph node metastasis and surgical strategy used to treat early gastric carcinoma. World J. Gastroenterol..

[27] Abe N (2002). Risk factors predictive of lymph node metastasis in depressed early gastric cancer. Am. J. Surg..

[28] Woo J (2004). Application of minimally invasive treatment for early gastric cancer. J. Surg. Oncol..

[29] Pyo J (2016). A risk prediction model based on lymph-node metastasis in poorly differentiated–type intramucosal gastric cancer. PLoS ONE.

